# Advancing precision, safety, and education in D3 lymph node dissection for right hemicolectomy using mixed reality technology

**DOI:** 10.1038/s41598-025-99961-2

**Published:** 2025-04-29

**Authors:** Shunjin Ryu, Yuta Imaizumi, Shunsuke Nakashima, Keisuke Goto, Hyuga Kawakubo, Hironari Kawai, Takehiro Kobayashi, Ryusuke Ito, Yukio Nakabayashi

**Affiliations:** Department of Digestive Surgery, Kawaguchi Municipal Medical Center, 180, Nishiaraijuku, Kawaguchi City, Saitama 333-0833 Japan

**Keywords:** Right hemicolectomy, Mixed reality, Colon cancer, Surgery, Intraoperative navigation, Gastroenterology, Oncology

## Abstract

Right hemicolectomy (RHC) is an important treatment for colorectal cancer. The superior mesenteric artery and superior mesenteric vein are known for their significant vascular variations. This study evaluated the short-term outcomes of integrating Mixed Reality (MR) technology into RHC for the treatment of colorectal cancer. Patients who underwent RHC for clinical stage II or III colon cancer between January 2015 and August 2024 were included. Patients were divided into two groups: the MR (+) group (n = 47), in which MR was used, and the MR (−) group (n = 145), in which MR was not used. MR using SYNAPSE VINCENT, Holoeyes MD, and HoloLens2 was utilized for detailed 3D visualization of the vascular anatomy preoperatively and intraoperatively. Forty-four patients per group were matched via propensity score matching and surgical outcomes were compared. In both groups, approximately 70% of the surgeries were performed by the training surgeon. Compared with the MR (−) group, intraoperative blood loss and hospital stay were decreased, and the number of lymph nodes harvested around the middle colic artery/vein were increased without prolonging the operative time in the MR (+) group. MR in RHC offers surgical precision, safety, enhanced patient recovery, and educational value.

## Introduction

Right hemicolectomy (RHC) is a fundamental procedure in the surgical management of colorectal cancer and is frequently performed by young physicians as part of their training. Data from the 2020 Japanese National Clinical Database (NCD) concerning RHC revealed that 54.2% of RHC procedures were performed laparoscopically, with a complication rate (Clavien–Dindo grade 3 or higher) of 8% and a mortality rate within 30 days postsurgery of 1.4%^1^. These statistics suggest that there is considerable room for improvement in the outcomes of this common procedure.

In recent years, the importance of complete mesocolic excision (CME) and central vascular ligation (CVL) in improving the prognosis of colon cancer surgeries has been increasingly recognized^2–10^. CVL is an essential component of the D3 dissection procedure, which has been performed in Japan to treat advanced colorectal cancer. These techniques, particularly when applied to the superior mesenteric artery (SMA) and superior mesenteric vein (SMV), are technically challenging and carry significant risks, including life-threatening hemorrhage. The increasing use of minimally invasive surgery (MIS), particularly laparoscopic procedures, has introduced new challenges in surgical education, as the tactile feedback and three-dimensional (3D) spatial awareness provided by open surgery are limited. The complexity of these procedures, combined with the need for precise anatomical knowledge, highlights the need for advanced training tools that can accelerate the learning curve without compromising patient safety. One of the factors complicating D3 lymph node dissection or CVL in RHC is the high variability in the branching patterns of the SMA and SMV^11–14^.

**Fig. 1:**
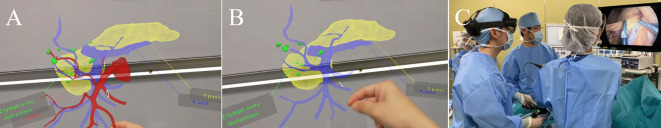
(**a**) Preoperative simulation with Mixed Reality: Surgeons manipulate the 3D hologram and confirm the vascular anatomy. (**b**) The transparency of each structure can be adjusted. For example, it is possible to remove arteries from a 3D hologram to focus primarily on observing veins. (**c**) Laparoscopic right hemicolectomy was performed while wearing a HoloLens2 and viewing a 3D hologram.

To address these challenges, we utilized SYNAPSE VINCENT (Fujifilm, Tokyo, Japan) to extract and reconstruct the vascular anatomy around the SMA and SMV from CT images. This detailed 3D vascular model was then integrated into a Mixed Reality (MR) environment via Holoeyes MD (Holoeyes, Inc., Tokyo, Japan) and viewed through transparent MR glasses, specifically HoloLens2 (Microsoft, Microsoft, Redmond, WA, USA)^15,16^. Since August 2021, this innovative MR surgical support system has been employed for RHC at our institution, allowing surgeons to visualize patient-specific anatomical structures preoperatively and in real time during surgery (Fig. 1).

A pilot study reported the feasibility of this innovative MR surgical support in colorectal cancer surgery^17^. The aim of this study was to analyze the short-term outcomes of integrating MR into surgical planning and execution of RHC for colorectal cancer.

## Methods

### Patients and methods

A total of 192 patients who underwent RHC between January 2015 and August 2024 for clinically diagnosed stage II or III (TNM classification 8th version) colon cancer, which requires D3 lymph node dissection oncologically, were divided into two groups: RHC in the MR(+) group (n = 47) was supported through the use of MR, whereas RHC in the MR(−) group (n = 145) was not MR-assisted. MR was employed for patients scheduled for RHC with D3 dissection post-August 2021. Since August 2021, the exclusion criterion for the MR (+) group included patients who could not undergo contrast-enhanced CT due to renal dysfunction or allergies. In the MR (−) group, 3D vascular model creation or intraoperative CT navigation was not utilized.

This single-center retrospective cohort study was approved by the Research Ethics Committee of the Kawaguchi Municipal Medical Center (Saitama, Japan) (approval number: 2021–19). Patients in the MR( +) group provided consent to participate in this study.

This investigation was conducted in accordance with the Declaration of Helsinki.

### Main outcomes

Patient background, surgeon’s experience, surgical approach, and short-term surgical outcomes were compared. Surgical outcomes included blood loss volume, operative duration, conversion to open surgery, length of postoperative stay, postoperative complications [Clavien‒Dindo grade III or above], operative mortality, the number of harvested lymph nodes, and the number of treated vessels.

Blood loss was estimated by measuring the weight of suctioned fluids and gauze before and after use. In cases where irrigation was performed, the irrigation volume was subtracted from the total suctioned volume to ensure accuracy.

Harvested lymph nodes were classified as follows: pericolic (No.201, 211, 221), intermediate (No.202, 212, 222), and main lymph nodes (No.203, 213, 223) along the ileocolic, right colic, and middle colic arteries and veins, respectively^18^. The number of harvested lymph nodes in each category, the total number of main lymph nodes, and the overall total number of lymph nodes were compared.

The number of treated vessels was counted based on intraoperative records. These vessels were defined as branch blood vessels from the SMA and SMV that were ligated for lymph node dissection with the anatomical recognition.

### Imaging and data processing

CT imaging was performed via the SOMATOM Definition FLASH (Siemens Healthineers, Tokyo, Japan), with contrast agent injected via the Dual Shot GX7 injector (Nemoto Kyorindo, Tokyo, Japan). The contrast imaging protocol employed a bolus tracking method, with monitoring at the level of the celiac artery. Arterial phase imaging was triggered when the CT value exceeded 150 HU, with a delay time of 6 s before acquisition in dual-energy mode. Portal venous phase imaging was performed 60–80 s postinjection, with slight delays manually adjusted to optimize arterial and venous visualization, considering factors such as patient breath holding. Imaging was conducted with a slice thickness of 0.6 mm, using a 20 G intravenous line in the right arm and a contrast agent dose of 540 mg/kg body weight, followed by a saline flush.

For each patient in the MR(+) group, the vascular anatomy branching from the SMA and SMV, pancreas, and lymph node metastases was extracted as individual DICOM datasets via SYNAPSE VINCENT. This process was partially conducted by radiological technologists, with adjustments made by the operating surgeons. Since our facility did not have the additional option in Synapse Vincent for converting 3D polygon data (referred to as surface rendering and surface editing) directly to the STL or OBJ formats), we utilized OsiriX (Newton Graphics, Sapporo, Japan) instead. The extracted DICOM data were then imported into OsiriX, where they were converted into STL- or OBJ-form 3D polygon data and subsequently uploaded to the Holoeyes MD system. Finally, the 3D polygon data were downloaded to HoloLens2, allowing the surgical team to interact with the 3D holograms as MR images preoperatively and during surgery.

### Preoperative simulation

Wearing HoloLens2, via MR, surgeons viewed 3D images of branches from the SMA and veins, pancreas, and lymph node metastasis. Specific attention was given to the following: ileocolic artery and vein, right colic artery and vein, middle colic artery and vein, Henle’s gastrocolic trunk, accessory right colic vein, and the first jejunal vein, as well as the location of lymph node metastases. These structures were visualized and analyzed in detail during preoperative planning.

### Intraoperative use of mixed reality

**Fig. 2:**
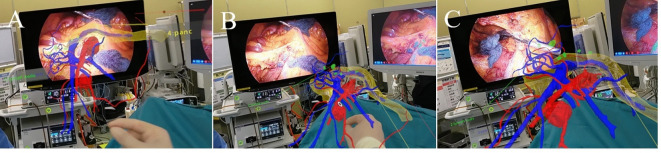
(**a**) A video recording of the surgeon’s view. A 3D hologram can be placed next to the laparoscopic monitor at any size and angle. Lymph node dissection in front of the superior mesenteric vein (SMV) during laparoscopic right hemicolectomy. (**b**) The angle of the 3D hologram is adjusted using the SMV as a landmark. (**c**) The right colic artery is dissected while the anatomy is confirmed via a 3D hologram.

The surgeons used MR images during surgery via HoloLens2. When it was necessary to confirm the vascular course, a 3D hologram was positioned next to the monitor and aligned with the surgical field, and surgery was conducted while verifying the vascular anatomy (Fig. 2).

HoloLens2 use was limited to the process of CVL, such as at the beginning of lymph node dissection and at the time of confirmation of vascular branches. Therefore, the HoloLens2 was not worn continuously throughout the surgery; it was removed when not in use, such as during bowel mobilization, specimen extraction, and anastomosis.

### Data analysis

Propensity scores were calculated for each patient using a logistic regression model based on the following covariates: sex, age, body mass index, tumor stage, extent of lymph node dissection, abdominal surgical history, surgical approach (laparoscopic or open surgery), and surgeon’s experience. We used one-to-one nearest neighbor matching without replacement, with a caliper width of 0.2 to adjust for baseline differences. The results are expressed as the median and interquartile range. Statistical analyses were performed with the Mann–Whitney U test and Fisher’s exact test, and all differences were determined to be significant at p < 0.05.

## Results

Patient characteristics, surgical details, and surgeon`s experience before propensity score matching are shown in Table 1.

**Table 1:** Comparison of patient characteristics, surgical details, and surgeon`s experience before propensity score matching.

Characteristics		MR ( +) (n = 47)	MR (−) (n = 145)	p value
Age (year)		77 [68–81]	76 [71–81]	0.8764
Sex	Man: Woman	29:18	77:68	0.3029
Body Mass Index		23.5 [20.7–25.5]	21.9 [19.2–24.4]	0.0413
Abdominal surgical history	History: no history	20:27	49:95	0.2908
Tumor location	Cecum	1 (2.1%)	13 (9.0%)	0.1654
	Ascending colon	36 (76.6%)	88 (60.7%)	
	Transverse colon	10 (21.3%)	42 (29.0%)	
	Ascending colon and transverse colon	0	2 (1.4%)	
T stage ^†^	1:2:3:4a:4b	1:8:30:7:1	4:18:97:16:10	0.6426
N stage ^†^	0:1a:1b:2a:2b	28:6:10:1:2	95:19:22:7:2	0.5541
M stage ^†^	0:1	47: 0	144: 1	1.000
D3 Lymph node dissection		46 (97.9%)	101 (69.7%)	< 0.0001
Laparoscopic surgery		47 (100%)	98 (67.6%)	< 0.0001
Surgeons with less than 4 years of experience in gastrointestinal surgery		30 (63.8%)	98 (67.6%)	0.6350

^†^Pathological confirmation according to the 8th Edition of the UICC TNM classification.

Values are presented as the medians [interquartile range] or n (%).

There were no significant differences in age, sex, tumor location, or TNM stage (TNM classification 8th) between the MR (+) group and the MR (−) group. Patients in the MR (+) group had a higher BMI than those in the MR (−) group. In the MR (+) group, the rates of laparoscopic surgery and successful D3 lymph node dissection were significantly greater than those in the MR (−) group.

Patient characteristics, details of surgery, and surgeon`s experience after propensity score matching are shown in Table 2.

**Table 2:** Comparison of patient characteristics, surgical details, and surgeon`s experience after propensity score matching.

Characteristics		MR ( +) (n = 44)	MR (−) (n = 44)	p value
Age (year)		77 [68–81]	74 [67–78]	0.2327
Sex	Man: Woman	27:17	25:19	0.6646
Body Mass Index		23.5 [20.6–25.6]	23.8 [20.3–25.3]	0.9867
Abdominal surgical history	History: no history	17:27	15:29	0.658
Tumor location	Cecum	0	5 (11.4%)	0.0508
	Ascending colon	35 (79.6%)	28 (63.6%)	
	Transverse colon	9 (20.5%)	9 (20.5%)	
	Ascending colon and transverse colon	0	2 (4.6%)	
T stage ^†^	1:2:3:4a:4b	1:8:27:7:1	1:7:32:4:0	0.6792
N stage ^†^	0:1a:1b:2a	28:5:10:1	31:4:9:0	0.7253
M stage ^†^	0:1	44: 0	44: 0	1.000
D3 Lymph node dissection		43 (97.7%)	42 (95.5%)	1.000
Laparoscopic surgery		44 (100%)	44 (100%)	
Surgeons with less than 4 years of experience in gastrointestinal surgery		30 (68.2%)	29 (65.9%)	0.8206

^†^Pathological confirmation according to the 8th Edition of the UICC TNM classification.

Values are presented as the medians [interquartile range] or n (%).

There was no significant difference in age, sex, BMI, abdominal surgical history, tumor location, TNM stage, surgeon`s experience, and rates of laparoscopic surgery and D3 lymph node dissection. Similar percentages of surgeries were performed by surgeons with less than 4 years of experience in both groups (68.2% in the MR (+) group vs. 65.9% in the MR (−) group, p = 0.8206).

Surgical outcomes are shown in Table 3.

**Table 3:** Comparison of surgical outcomes after propensity score matching.

Outcomes	MR( +) (n = 44)	MR(-) (n = 44)	p value
Conversion to open surgery	0	3 (6.8%)	0.2414
Blood loss (ml)	3 [1–5]	10 [5–39]	< 0.0001
Operation time (minute)	289 [253–325]	298 [232–386]	0.7072
Post-operative hospital stay (days)	8 [8–10]	9 [8–12]	0.003
Post-operative complication (Clavien‒Dindo grade III or above)	3 (6.8%)	2 (4.6%)	1.000
Operative mortality ^†^	0	0	1.000
The number of treated vessels	5 [4.25–6]	5 [3–5]	0.0159
Number of harvested lymph nodes			
No.201, pericolic lymph nodes for the ICAV	6.5 [4–12]	8 [5–12]	0.2415
No.202, intermediate lymph nodes for the ICAV	4 [3–7]	5 [3–7]	0.4397
No.203, main lymph nodes for the ICAV	4 [2–6]	4 [2–5]	0.5701
No.211, pericolic lymph nodes for the RCAV	4 [2.5–6.25]	4 [1–7]	0.9355
No.212, intermediate lymph nodes for the RCAV	2 [1.5–3.5]	3 [2–4.25]	0.1838
No.213, main lymph nodes for the RCAV	3 [2–4]	3 [2–4.25]	0.7872
No.221, pericolic lymph nodes for the MCAV	3.5 [1.75–5]	4 [1–6]	0.8509
No.222, intermediate lymph nodes for the MCAV	3.5 [2–6.25]	3 [2–5]	0.4432
No.223, main lymph nodes for the MCAV	3 [2–6]	2 [0–4]	0.0016
Total main lymph nodes (203, 213, 223)	8 [5–11]	5.5 [3–9]	0.0205
Total lymph nodes	33.5 [25.25–41]	27.5 [21.25–37.5]	0.1941

Notes:

No 201,202,203,211,212,213,221,222,223; These classifications are based on the Japanese guidelines. ICAV; ileocolic artery and vein, RCAV; right colic artery and vein, MCAV; Middle colic artery and vein.

^†^Operative mortality is a combination of 30-day mortality and death during hospitalization 31–90 days after surgery.

^‡^8th Edition of the UICC TNM classification.

Values are presented as the medians [interquartile range] or n (%).

In the MR (+) group, blood loss was lower, and the postoperative hospital stay was shorter significantly than in the MR (−) group. The rate of conversion to open surgery was 0% in the MR (+) group and 6.8% in the MR (−) group. Although the difference was not statistically significant, there was a trend towards a lower conversion rate in the MR (+) group. There was no difference in operation time.

The rate of Clavien-Dindo complications ≥ grade 3 was 6.8% in the MR (+) group (1 incisional hernia requiring reoperation and 2 bowel obstruction treated with a long tube), and 4.6% in the MR (−) group (1 anastomotic leakage requiring reoperation and 1 anastomotic leakage requiring percutaneous drainage), the difference of which was not statistically significant.

The number of treated vessels was higher in the MR (+) group than in the MR (−) group. Regarding harvested lymph nodes, the number of No.223 lymph nodes, which are the main lymph nodes for the middle colic artery and vein, was only higher in the MR (+)group.

## Discussion

The usefulness of surgical preparation in understanding the detailed vascular anatomy via 3D rendering from contrast-enhanced CT has been reported^19,20^. Compared with a formal CT interpretation method, 3D models improve the anatomical understanding of mesenteric vascular anatomy in surgical trainees^21^. However, these are merely 3D-like images viewed on a 2D monitor. Some studies report that only people with high spatial awareness are able to exploit the complex information in 3D images on 2D monitors^22^. There are few reports on surgical support that allows surgeons to observe the vascular anatomy in true 3D using MR technology during surgery^23,24^. This study highlights the significant advantages of using MR technology in RHC, particularly for challenging Japanese D3 dissections around the superior mesenteric artery and vein. The use of SYNAPSE VINCENT and Holoeyes MD allows for the creation of precise 3D models of patient-specific anatomy, which can be visualized beside the laparoscopic monitor in real time during surgery through devices such as HoloLens2. This technology addresses several key challenges in modern colorectal surgery, including the difficulty of mastering laparoscopic techniques, the risk of severe hemorrhage during CVL, and the need for enhanced educational tools for training young surgeons.

The following points of anatomical structures were particularly carefully confirmed with MR both preoperatively and intraoperatively:

1. The flow pattern of the ileocolic vein: Does it flow directly into the SMV, form a common trunk with the right colic vein and then flow into the SMV, or flow into the gastrocolic trunk (GCT)?
2. The positional relationship between the ileocolic artery and ileocolic vein.
3. The presence of the right colic artery and right colic vein and their distance from the ileocolic artery and vein, respectively.
4. The presence and number of accessory right colic veins entering the GCT.
5. Do the right and left branches of the middle colic artery and vein independently drain into the SMA and SMV, respectively, or do they merge to form a common trunk?
6. Does the middle colic vein form a common trunk with the inferior mesenteric vein or the first jejunal vein?
7. Is the first jejunal vein dorsal or ventral from the SMA? Is it cranial or caudal from the root of the middle colic artery?
8. The positional relationship between the inferior border of the pancreas and the root of the middle colic vein.

It is difficult to remember and pay attention to all of this information, specifically and in detail, when performing surgery.

Studies have shown that 3D methods have a positive effect on human memory and that, compared with traditional or 2D digital methods, 3D methods with virtual reality can potentially improve the effectiveness of teaching anatomy^25,26^.

The following four positive effects of 3D on memory are expected.

1. **Memory Enhancement:** Compared with 2D images, 3D images and models provide visually more realistic and three-dimensional information, which can leave a stronger impression. This is believed to make it easier for information to be stored in long-term memory.
2. **Improvement of Spatial Awareness:** 3D environments and models allow for a more intuitive understanding of spatial relationships and positions, making them particularly effective in learning complex anatomical structures or spatial tasks.
3. **Realism and Engagement:** 3D content is visually appealing and enhances user engagement, which in turn increases learning motivation and concentration, leading to better retention of information.

**Fig. 3:**
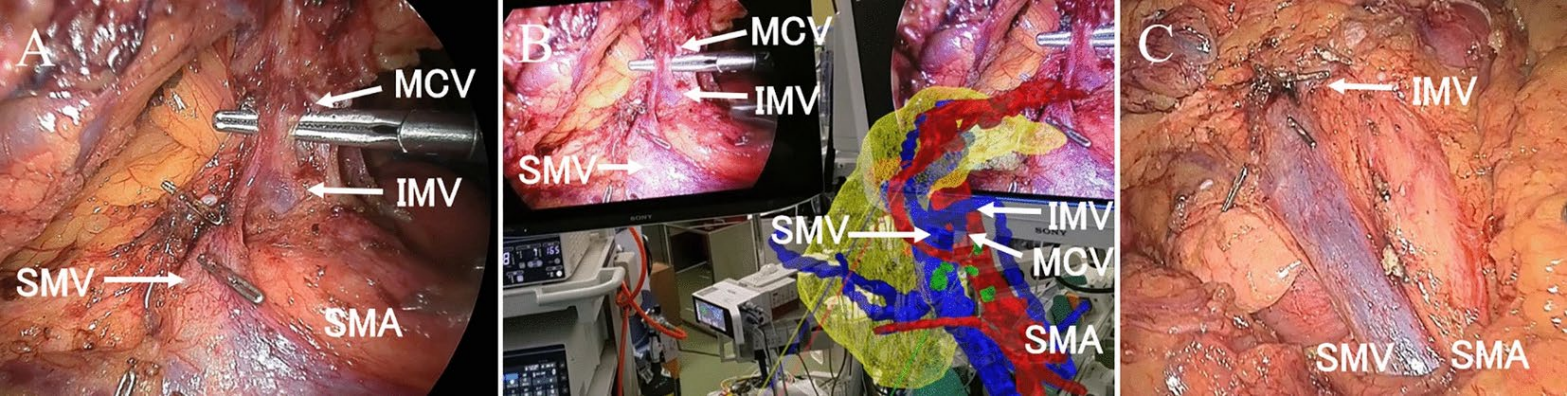
(**a**) The tissue in front of the superior mesenteric artery (SMA) and superior mesenteric vein (SMV) is dissected up to the lower border of the pancreas. The middle colic vein (MCV) is dissected such that the inferior mesenteric vein (IMV) flow into the MCV is preserved. (**b**) The hologram shows that the MCV and IMV form a common trunk and drain into the superior mesenteric vein (SMV). Surgery was performed while the hologram was viewed. (**c**) Lymph node dissection and central vascular ligation were performed to preserve the IMV.

**Fig. 4:**
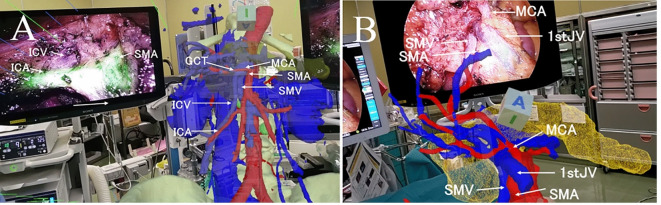
(**a**) A rare case where the ileocolic vein (ICV) drains into the superior mesenteric vein (SMV) via the gastrocolic trunk (GCT). The ICV runs such that it appears to approach near the root of the ileocolic artery obliquely from the cranial side of the surgical field. MCA: middle colic artery, SMA: superior mesenteric artery. (**b**) Rare case in which the first jejunal vein (1st JV) passes in front of the SMA, caudal to the middle colic artery. If this information is not considered during dissection of the anterior surface of the SMA, there is a risk of damage to the 1st JV, which could lead to dangerous bleeding.

The most useful aspect of MR is that it allows surgeons to place a 3D hologram next to the laparoscopic monitor and adjust the angles of the SMV and SMA to match the surgical field, allowing the surgeon to intuitively confirm the anatomy while proceeding with the surgery—much like adjusting a map’s orientation as you walk or drive (Figs. 3 and 4).

Furthermore, the transparency of each structure can be adjusted so that, for example, only veins can be observed.

When dissecting fat tissue from the caudal side of the SMV and the SMA toward the inferior border of the pancreas, it is crucial to obtain a 3D sense of depth and distance to understand the spatial relationship until the next vascular branch appears.

Attempts to superimpose a 3D hologram over the operative field in laparotomy have been reported^27^. The MR-assisted surgical support conducted in this study does not involve the use of a tracker to overlay images onto the surgical field. Image overlay on the surgical field could interfere with the detailed local anatomical observation achieved through the magnification effect of laparoscopy; therefore, it is considered unnecessary for laparoscopic RHC.

MR has two major limitations. The HoloLens 2 weighs 566 g, and the field of view appears relatively dim when wearing it. The development of lightweight MR glasses that provide an optimal surgical view is needed. Another limitation concerns the mobility of vessels during surgery. If vessels branching from the SMA and SMV are displaced due to bowel mobilization or dissection, the 3D hologram does not dynamically adjust to reflect these changes. To achieve real-time tracking and adaptation of the 3D hologram to vessel movement, intraoperative CT imaging in a hybrid operating room and subsequent reconstruction of the 3D hologram from the acquired images are required. However, considering the time and effort involved, this approach is currently impractical for routine clinical use.　Nevertheless, the critical anatomical information for lymph node dissection and central vascular ligation (CVL) lies at the vascular root rather than the position of peripheral vessels distant from the root. As a result, this limitation does not significantly diminish the clinical usefulness of MR-assisted navigation in D3 lymph node dissection.　To enhance the adaptability of Mixed Reality technology in surgery, future developments should focus on real-time updates of 3D holograms. Possible advancements may include AI-assisted intraoperative image processing, integration with augmented reality-based tracking systems, and enhanced real-time vascular segmentation techniques. These innovations could improve the accuracy of MR guidance, making it a more dynamic and responsive tool for complex colorectal surgeries.

In this study, according to patient background, before matching, some patients underwent D2 lymph node dissection instead of D3 dissection, even though the cancer was advanced. The reason for this unusual treatment is that the actual extent of lymph node dissection was determined based on the attending surgeon’s clinical judgment, considering patient-specific factors such as age, comorbidities, surgical findings, and overall surgical risk. In the MR (–) group, the D3 lymph node dissection rate was 69.7%, and the laparoscopic surgery rate was 67.6%.

While the laparoscopic surgery rate was comparable to national data, the variability in lymph node dissection reflects real-world clinical practice in general hospitals, where treatment decisions are individualized to balance oncologic benefit and surgical safety^1^.

After matching, including those who underwent laparoscopic surgery and considering the extent of lymph node dissection, the introduction of MR allows reduced blood loss and length of postoperative hospital stay in patients with RHC undergoing D3 lymph node dissection. Furthermore, in the MR ( +) group, the number of blood vessels ligated for lymph node dissection and the number of harvested main lymph nodes of the middle colic artery and vein were increased without prolonging the operative time. These findings support the fact that MR allows for a three-dimensional understanding and memory of vessel anatomy, significantly enhancing the surgeon’s ability to perform precise dissections. Because we could anticipate the branching points of blood vessels or unusual anatomical points during dissection, the incidence of unnecessary vascular injuries was reduced. Vascular injuries sometimes require conversion to open surgery or conversion to D2 lymph node dissection because intraoperative safety takes precedence over oncologic requirements. The reduction in blood loss was thought to have contributed to achieving D3 lymph node dissection with no conversions to open surgery. These positive factors accelerated postoperative recovery and significantly reduced the length of postoperative hospital stay. According to a multicenter prospective observational study conducted at five specialist cancer hospitals in Japan, the metastasis rates in transverse colon cancer were reported as 7.5% around the middle colic vein and 2.5% around the middle colic artery, indicating a high prevalence of lymph node metastasis at the root of the middle colic vessels^28^. Based on these findings, MR, which improved the number of harvested No.223 lymph nodes around the middle colic artery and vein, may enhance the precision of lymph node dissection and contribute to better long-term patient outcomes.

Furthermore, in the MR (+) group, approximately 70% of surgeries were performed by surgeons with limited clinical experience. Because the pathways for lymph node dissection and central vascular ligation are visualized in a 3D hologram like a map, the difficulty of surgery was reduced, and the frequency of surgeries performed by junior surgeons was maintained without decreasing the quality of surgeries.

Considering this, it is reasonable to expect that MR could significantly contribute to safe surgical education.

## Conclusions

MR-assisted surgical support during RHC, particularly applied to D3 lymph node dissection around the SMA and SMV, provides significant benefits by increasing the number of No.223 lymph nodes harvested, which may contribute to improved long-term oncologic outcomes. Its integration with 3D imaging enhances the precision of lymphadenectomy, leading to reduced intraoperative blood loss, shorter hospital stay, and increased safety in surgical training for young surgeons. Given these advantages, MR technology should be considered a valuable tool for optimizing both patient outcomes and surgical education.

### Limitations

The limitations of this study include its retrospective design and small sample size.

The utility of MR depends on the accuracy of the created 3D hologram. To improve the accuracy of vascular anatomy extraction via SYNAPSE VINCENT, a CT scan with 0.6 mm slices was used, and the timing of contrast enhancement was manually adjusted. Nonetheless, importantly, the vascular anatomy viewed through MR may be inaccurate. Surgeons should not blindly trust the vascular anatomy presented by MR but must recognize its potential inaccuracies.　It is crucial not to perform dissection in an inadequately exposed surgical field or when tissue tension is insufficient but to always use surgical techniques that minimize the risk of accidental vascular injury, regardless of where vessels may suddenly appear.

## Data Availability

The data that support the findings of this study are available from the corresponding author upon request.
